# A Special Issue Dedicated to the 110^th^ Anniversary of Henan University

**DOI:** 10.1002/EXP.20220101

**Published:** 2022-08-31

**Authors:** Jia Li, Shanhu Liu, Chun‐Peng Song

**Affiliations:** ^1^ School of Pharmacy Henan University Kaifeng Henan China; ^2^ College of Chemistry and Chemical Engineering Henan University Kaifeng Henan China; ^3^ State Key Laboratory of Crop Stress Adaptation and Improvement, School of Life Sciences Henan University Kaifeng Henan China

The year 2022 marks the 110^th^ anniversary of Henan University. To celebrate this special event, it is our great privilege to present this Special Issue of *Exploration* to showcase the recent research progress in interdisciplinary science led by the faculty and alumni from Henan University. We hope that this Special Issue will provide a unique opportunity to demonstrate the excellent research and vision of the authors.


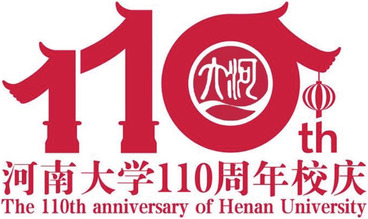


Established in 1912 as “the Preparatory School for Further Study in Europe and America” Henan University was then one of the three training bases for studying abroad in China. In 2008, it became a co‐construct university by Henan Province and the Ministry of Education of China, a “Plan 111” construction university in 2016, and “Double First‐Class” initiative university in 2017. Currently, Henan University has a strong faculty of over 4600, including 22 academicians working full time and part time, as well as over 1700 professors and associate professors. 50,000 full‐time students, including 12,000 graduate students and over 1200 international students. With strong support from the central and provincial governments, faculties and students, Henan University is on the pathway to becoming a world first‐class university.

This Special Issue features 6 *Research Articles and 4 Reviews*, with a focus on materials for drug delivery and treatment, disease diagnosis, energy, water treatment and crystal materials. For drug delivery, Shi *et al.* from Henan University employed *ApoE* peptide to decorate erythrocyte membrane camouflaged nanoparticle, which greatly enhanced blood circulation time and blood–brain barrier penetration. Through encapsulating, this nanoparticle successfully co‐delivered temozolomide (TMZ) and the epigenetic bromodomain inhibitor OTX015 to glioblastoma and achieve an excellent therapeutic effect, providing a potential new treatment for this terrible disease; Yan *et al*. from Chinese Academy of Sciences and Zhengzhou University developed a single‐atom nanozyme with a divalent Cu‐N4 structure (Cu‐SAzyme) to mimic the electronic and structural features of natural Cu‐only SOD5, which showed high SOD‐like activity and good stability at a wide catalytic pH and temperature range. More importantly, Cu‐SAzyme significantly eliminated excess ROS and pro‐inflammatory cytokines in activated inflammatory cells, and effectively reduced multiple organ damage and mortality in septic animals. Nanomaterials with the capability for both imaging and treatment show promising applications. Xie *et al*. from Changchun Institute of Applied Chemistry and University of Science and Technology of China developed an indocyanine green (ICG)‐modified delivery nanoplatform, which promotes the formation of paclitaxel dimer, leading to high drug content and controlled drug release in the targeted tumor sites. In addition, the near‐infrared fluorescence character of ICG allowed real‐time imaging of drug distribution in vivo, making it possible to integrate treatment and imaging. For disease diagnosis, quantum dots (QDs) are promising fluorescent probes. However, the state‐of‐the‐art CdSe QDs are potentially harmful to living organisms due to the heavy metal Cd ions. To overcome this shortcoming, Shen *et al.* from Henan University developed Cd‐free QDs, named InP QDs, which showed great biocompatibility. InP QDs can be used to detect alpha‐fetoprotein, a serum marker of hepatocellular carcinoma in a wider range with a lower limit of detection than CdSe QDs. It can also specifically label liver cancer cells in animal models, making it a great candidate for cancer diagnosis and image‐guided surgery.

As representative examples of energy research, Liu *et al.* from Donghua University and Jiangnan University developed a hydrogel complex that consists of the self‐wrinkled polyaniline (PANI)‐based composite hydrogel (SPCH) with an electrolytic hydrogel and a PANI composite hydrogel. In this complex, PANI‐based hydrogel exhibited large stretchability and high fatigue resistance due to its self‐wrinkled surfaces and the intrinsic stretchability of hydrogels, paving the way to develop conductive polymer‐based hydrogels for all‐in‐one stretchable supercapacitors with large deformation tolerance and high energy density. In the field of energy, this issue also provides an excellent overview, authored by Zhao *et al.* from Henan University, on the challenges and advanced strategies to boost the electrochemical performance of redox‐active organic electrode materials (OEMs) for sustainable secondary batteries. Particularly, the characterization technologies and computational methods to elucidate the complex redox reaction mechanisms and confirm the organic radical intermediates of OEMs have been introduced. For environment protection, Jiang *et al.* from Qingdao Institute of Bioenergy and Bioprocess Technology developed a forward‐osmosis (FO) and photothermal evaporation (PE) coupling system (FO‐PE) for continuous FO separation with a steady water flux, a challenge remaining under continuous operation. Furthermore, the solar‐powered FO‐PE coupling system makes it significantly meaningful for practical applications. Also related to water treatment, Li *et al.* from the Qingdao Institute of Bioenergy and Bioprocess Technology and University of Chinese Academy of Sciences summarized the recent progress in biofibrous nanomaterials, including cellulose nanofibrils, chitin nanofibrils, and protein nanofibrils, which have shown great advantages in capturing metal ions from water. In particular, the advancement in the extraction of typical strategic metal ions such as noble metal ions, nuclear metal ions, and Li‐battery related metal ions is reviewed. Cheng *et al.* from Beihang University and Zhengzhou University reviewed the latest research on MXene‐based nanocomposite film, a new type of two‐dimensional nanomaterials that has been extensively used for energy storage, water treatment, lubrication, and catalysis. The authors focused on the fabrication approach, the mechanical properties, and the applications of MXene‐based nanocomposite films. As representative samples of crystal materials, Xue *et al.* from Shenzhen Institute of Advanced Technology reviewed the preparation and characterization Lithium niobate (LN), a type of multifunctional dielectric and ferroelectric crystal that is widely used in acoustic, optical, and optoelectronic devices. This review outlines the advanced methods used to characterize both the composition and homogeneity of LN melts and crystals from the micro‐ to wafer scale.

We appreciate the supports and contributions from the authors to this Special Issue. We sincerely hope that this Special Issue will promote collaborations between Henan University and institutes around the world.

